# Implementing a nationwide criteria-based emergency medical dispatch system: a register-based follow-up study

**DOI:** 10.1186/1757-7241-21-S2-A31

**Published:** 2013-09-09

**Authors:** Mikkel S Andersen, Søren Paaske Johnsen, Jan Nørtved Sørensen, Søren Bruun Jepsen, Jesper Bjerring Hansen, Erika Frischknecht Christensen

**Affiliations:** 1 Research Department, Prehospital Emergency Medical Services, Aarhus, Central Denmark Region, Denmark; 2 Dept. of Clinical Epidemiology, Aarhus University Hospital, Denmark; 3 Emergency Medical Communication Center, Capital region of Denmark, Denmark; 4 Emergency Medical Communication Center, Odense University hospital, Region of Southern Denmark, Denmark

## Background

The organization of prehospital care in Denmark has recently been fundamentally revised. All 112 calls concerning illness and injury are now redirected to one of five emergency medical communication centers (EMCCs), staffed with nurses, paramedics and doctors. Assessment of 112 callers with medical problems has up until now, been conducted mainly by the police. The EMCC staff uses a priority dispatch protocol (The Danish Index for Acute Care) to divide all callers into five levels acuity (A-E), level A being patients with potential life-threatening symptoms.

We present the first data on mortality, admission rate and level of acuity of patients after implementation of emergency medical dispatch in Denmark.

## Methods

A follow-up study conducted in the tree largest regions in Denmark, representing 75 % of the Danish population. During a six months period, all 112 callers in contact with an EMCC where included in the study. Information on vital status and hospital contacts where obtained through national population-based registries.

## Results

In total we identified 99,855 contacts to the EMCC via the 112 alarm number. 67,135 had information registered sufficiently for further investigation. 51.4% was assessed as acuity level A, 46.3% as B, 2.1% as C, 0.2% as D (level E not included). The case fatality rate for acuity level A patients on the same day as the 1-1-2 call was 4.4% (95% CI=4.1-4.6). This case-fatality rate was 14.3-fold (95 % CI=11.5-17.9) higher than for acuity level B–D patients. The hospital admission rate for acuity level A patients was 64.4% (95% CI=63.8-64.9). There was a significant trend (p<0.001) towards lower admission rates for patients with lower levels of acuity.

## Conclusion

The majority of patients were assessed as acuity level A or B according to the Danish Index for Acute Care. Case fatality and hospital admission rates were substantially higher for acuity level A patients than for acuity level B–D patients. Using case fatality and hospital admission rates as indicators of case severity, the newly implemented Danish criteria-based dispatch system appears to effectively triage patients according to the severity of their condition.

